# Assessing the therapeutic potential of long-chain isomaltooligosaccharides in diabetic and hyperlipidemic rats

**DOI:** 10.1186/s13098-024-01374-0

**Published:** 2024-07-16

**Authors:** Onrapak Reamtong, Rattiya Waeonukul, Pattaneeya Prangthip

**Affiliations:** 1 Department of Molecular Tropical Medicine and Genetics, Faculty of Tropical Medicine, Mahidol University, Bangkok, Thailand; 2 Excellent Center of Enzyme Technology and Microbial Utilization, Pilot Plant Development and Training Institute (PDTI), King Mongkut’s University of Technology Thonburi (KMUTT), Bangkok, 10150 Thailand; 3 Division of Biochemical Technology, School of Bioresources and Technology, King Mongkut’s University of Technology Thonburi (KMUTT), Bangkok, 10150 Thailand; 4 Department of Tropical Nutrition and Food Science, Faculty of Tropical Medicine, Mahidol University, Bangkok, 10400 Thailand

**Keywords:** *Bacillus subtilis* strain AP-1, Diabetes, Isomaltooligosaccharides

## Abstract

**Background:**

The global rise in diabetes prevalence necessitates effective treatments. Rats, mimicking physiological changes seen in Type 2 diabetes, serve as valuable models for studying metabolic disorders. Natural health supplements, especially prebiotics, are gaining interest for improving metabolic health. Isomaltooligosaccharides (IMOs), classified as functional oligosaccharides and prebiotics, have attracted attention due to their beneficial effects on gut microbiota balance and cholesterol reduction. However, commercial IMOs often contain undesirable sugars, leading to the development of long-chain IMOs with enhanced prebiotic properties.

**Methods:**

This study assessed the therapeutic potential of long-chain IMOs derived from *Bacillus subtilis* strain AP-1 compared to inulin, a widely recognized prebiotic, in addressing hyperglycemia and hyperlipidemia in rats.

**Results:**

IMOs treatment effectively reduced blood sugar and triglyceride levels similarly to inulin supplementation. Proteomic analysis revealed changes in hepatic protein profiles, with upregulated pathways including glutathione metabolism, oxidative phosphorylation, and pentose and glucuronate interconversion, while pathways related to fatty acid and amino acid biosynthesis exhibited downregulation. These results suggest promising therapeutic effects of IMOs treatment on diabetes and hyperlipidemia by influencing key metabolic pathways.

**Conclusions:**

Our findings highlight the potential of long-chain IMOs as targeted interventions for metabolic disorders, warranting further investigation into their clinical applicability and mechanisms of action.

**Supplementary Information:**

The online version contains supplementary material available at 10.1186/s13098-024-01374-0.

## Introduction

The global prevalence of diabetes continues to rise annually, accounting for 95% of all diabetes cases worldwide [1]. Individuals with diabetes frequently encounter complications arising from elevated blood sugar and fat levels, heightening the risk of cardiovascular disease. Rats serve as prevalent models for investigating diabetes and other chronic conditions due to their parallel pathophysiological changes, including elevated insulin and blood sugar levels [2]. Elevated blood sugar levels trigger the production of detrimental oxidant agents such as reactive oxygen species and reactive nitrogen species, precipitating oxidative stress. This stress plays a pivotal role in the onset of vascular complications associated with diabetes, contributing to diabetic mortality [3]. In recent years, the research and development of health-promoting products have gained substantial momentum. Natural health supplements have garnered attention not only for their intrinsic nutritional value but also for their potential health benefits [4]. The global market for nutraceuticals and supplements has witnessed consistent growth over the past decade, reaching an estimated value of nearly $353 billion USD in 2019 [5]. Simultaneously, research in this domain has thrived, with over 70,000 articles published on PubMed between 2010 and 2020 focusing specifically on nutraceuticals or dietary supplements [4].

Among the myriad of dietary supplements derived from nature, prebiotic dietary fibers hold notable prominence. Prebiotics are defined as ingredients that undergo selective fermentation in the gastrointestinal tract, inducing specific changes in the composition and/or activity of the gut microbiota, thereby benefiting the host's health. These substances offer several advantages, including the improvement of gut barrier function and host immunity, reducing potentially harmful bacterial subpopulations, and augmenting the production of short-chain fatty acids (SCFAs). Prebiotics have demonstrated the ability to significantly increase the abundance of probiotics in fecal microbiota even at relatively modest consumption levels [6]. Moreover, the benefits of prebiotics have been extensively studied and researched in various physiological systems, including the digestive and immune systems, as well as in mitigating blood sugar and fat levels [7].

Isomaltooligosaccharides (IMOs) are categorized as functional oligosaccharides and prebiotics, composed of glucosyl saccharides featuring α-D-(1,6) linkages in the backbone, along with or without α-D-(1,4), (1,3), and (1,2) linkages [8, 9]. IMOs resist digestion by gastrointestinal enzymes, resulting in a low calorific value and glycemic index [10], and exhibit high stability under food processing conditions characterized by low pH and moderate temperatures [11]. Due to their diverse bioactivities, including promoting bifidobacteria proliferation, enhancing intestinal flora balance, facilitating vitamin synthesis, aiding mineral absorption, and reducing cholesterol levels, IMOs find extensive application as nutritional supplements, food ingredients, and beverages [12]. The health-promoting attributes of IMOs have been substantiated through both in vitro and in vivo investigations. IMOs assist in lowering blood sugar and triglyceride levels, improving intestinal structure, fostering the growth of beneficial gut bacteria, and mitigating the risk of colon cancer [9]. One particularly promising avenue involves utilizing IMOs to address diabetes and its associated complications, representing a significant public health concern worldwide. Commercially produced IMOs are commonly derived from starch sourced from cereal crops. These IMOs, which encompass short-chain varieties such as isomaltose and isomaltotriose, are purportedly absorbed in the small intestine. Nevertheless, they frequently contain surplus glucose and sugars, leading to fluctuations in blood sugar levels following consumption [13–15]. In contrast, long-chain IMOs appear to offer more benefits. Research indicates that they support probiotic growth and contribute to improved health more effectively [16–18]. Recently, a novel method of producing long-chain IMOs has emerged. These IMOs, derived from maltose via fermentation, exhibit promising prebiotic properties. They resist digestion in the gut, fostering the proliferation of beneficial bacteria in the colon. This process results in the production of beneficial SCFAs while discouraging the growth of harmful bacteria [19].

In this study, we investigated the potential health benefits of a novel series of long-chain IMOs derived from *Bacillus subtilis* strain AP-1, contrasting their effects with those of inulin, on hyperglycemia and hyperlipidemia in rats. Inulin is widely recognized as a beneficial prebiotic renowned for its resistance to digestion within the human gastrointestinal tract. Several reports have demonstrated that inulin effectively improves blood sugar control in individuals with diabetes and prediabetes, stimulating immune responses, and reducing levels of serum cholesterol, triacylglycerols, and phospholipids [20]. Additionally, this study integrates proteomic analysis to compare protein profiles between untreated diabetic and hyperlipidemic rats and those subjected to IMO treatment. By concentrating on specific analysis, we aimed to unravel the molecular mechanisms underlying the potential therapeutic effects of IMOs on diabetes and hyperlipidemia. Such insights hold promise for the development of targeted interventions tailored to individuals grappling with metabolic issue.

## Method

### Preparation of IMOs from maltose by *B. subtilis* strain AP-1

IMOs from *B. subtilis* strain AP-1 were prepared using the method described previously (Tiangpook et al., 2023). Initially, *B. subtilis* strain AP-1 was cultured on nutrient agar at 37 °C under aerobic conditions. To generate the inoculum, a single colony of *B. subtilis* strain AP-1 was selected and transferred to nutrient broth. The strain was then cultured at 37 °C with agitation at 200 rpm for 24 h until the optical density at 600 nm reached approximately 0.6, corresponding to a cell concentration of around 10^8^ colony-forming units per milliliter. For subsequent inoculation, 10% (v/v) of the *B. subtilis* strain AP-1 inoculum was subcultured onto Berg’s mineral salt fermentation medium enriched with 50 g/L of maltose at 37 °C with continuous agitation at 200 rpm for 36 h. Subsequently, samples were collected and subjected to heating at 100 °C for 10 min to deactivate any enzymatic activity, followed by centrifugation at 10,000 g for 10 min. The supernatant containing the IMOs was isolated using hollow-fiber ultrafiltration with a 10 kDa molecular weight cut-off membrane (GE Healthcare Bio-Sciences Corp., MA, USA). This ultrafiltration step effectively separated the IMOs from other compounds with molecular weights exceeding 10 kDa.

Subsequently, to enhance purity and remove color and odor, the recovered IMOs within the permeate fraction underwent treatment with activated carbon. This treatment involved agitating the permeate fraction with food-grade activated carbon (particle size ASTM 8 × 3, Master, Thailand), followed by filtration to remove the carbon. The treated IMOs were then recovered from the solution and subjected to freezing and subsequent lyophilization using a laboratory freeze dryer (FD5-T-Series; SIM International Group Co. Ltd., Newark, DE, USA). The purity of the IMOs product was determined by hydrolyzing the IMOs with acid and measuring the resulting glucose content using high-performance liquid chromatography (HPLC, Japan) equipped with an Aminex-87P column (Bio-Rad, USA) and detected by a refractive index detector, following the NREL Chemical Analysis and Testing Standard Procedure. detector (Shimadzu RID-10A, Japan) at 85 °C.

### Matrix-assisted laser desorption/ionization time-of-flight mass spectrometry (MALDI-TOF/MS)

Samples were prepared using the dried droplet method. A matrix solution of 10 mg/mL 2,5-dihydroxybenzoic acid (DHB) in 50% aqueous acetonitrile was mixed 1:1 (v/v) with the oligosaccharide sample solution. 1–2 μL of this mixture was spotted onto the MALDI target plate and allowed to dry, forming co-crystals. The dried sample spots were analyzed on a JMS-S3000 SpiralTOF^™^ MALDI-TOF/MS instrument (JEOL, Tokyo, Japan) at 20 kV accelerating voltage, using a 337 nm nitrogen laser for desorption/ionization. Mass spectra were acquired in the appropriate m/z range for oligosaccharides. The mass spectra were processed and analyzed using the SpiralTOF™ Series Complement software (JEOL). Oligosaccharide molecular ion peaks were identified based on their m/z values and isotopic patterns.

### Rat diet preparation

The ingredient proportions were adjusted as described previously [21] (Table 1). Inulin or IMOs were administered to the experimental rats by substituting cellulose fiber at a specific percentage of the total food weight for each assigned group. The food was provided to the rats daily at 10:00 am.

**Table 1:** Composition of diets in experimental groups

Ingredients	1. N	2. HF	3. DM	4. DM + 1% inulin	5. DM + 3% inulin	6. DM + 1% IMOs	7. DM + 3% IMOs
Casein (%)	20.0	20.0	20.0	20.0	20.0	20.0	20.0
Corn starch (%)	15.0	15.0	15.0	15.0	15.0	15.0	15.0
Sucrose (%)	50.0	33.0	33.0	33.0	33.0	33.0	33.0
Fiber (%)	5.0	5.0	5.0	4.0	2.0	4.0	2.0
DL- Methionine (%)	0.3	0.3	0.3	0.3	0.3	0.3	0.3
Mineral Mixture (%)	3.5	3.5	3.5	3.5	3.5	3.5	3.5
Vitamin Mixture (%)	1.0	1.0	1.0	1.0	1.0	1.0	1.0
Choline Bitartrate (%)	0.2	0.2	0.2	0.2	0.2	0.2	0.2
Corn Oil (%)	5.0	5.0	5.0	5.0	5.0	5.0	5.0
Lard (%)	–	17.0	17.0	17.0	17.0	17.0	17.0
Inulin (%)	–	–	–	1	3.0	–	–
IMOs (%)	–	–	–	–	–	1.0	3.0
Total (%)	100.0	100.0	100.0	100.0	100.0	100.0	100.0
Calorie in 100 g	185	388	388	388	388	388	388

All values are expressed as percentages unless otherwise indicated

*N* Rat groups receiving normal diets, *HF* Rat groups receiving high-fat diets, *DM* Rat groups induced with diabetes and receiving high-fat diets, *DM* + *1% inulin* Diabetic-induced rat group administered with 1% inulin and high-fat diets, *DM* + *3% inulin* Diabetic-induced rat group administered with 3% inulin and high-fat diets, *DM* + *1% IMOs* Diabetic-induced rat group administered with 1% IMOs and high-fat diets, *DM* + *3% IMOs* Diabetic-induced rat group administered with 3% IMOs and high-fat diets

### Experimental design

A total of 84 male rats, weighing approximately 180–220 g and aged 5 weeks, were acclimatized for 7 days with unrestricted access to standard rat food. Following acclimatization, the rats were randomly allocated based on body weight into two initial groups: the normal diet group (n = 12), which continued on a normal diet throughout the study, and the high-fat diet group (n = 72), which received a high-fat diet for 14 days.

Subsequently, the high-fat diet group was further subdivided based on body weight into two subgroups: a high-fat diet group (n = 12) and a diabetes group (n = 60). The 60 diabetic rats were induced to develop prediabetes using streptozotocin (STZ), which selectively damages pancreatic beta cells. STZ was administered via intraperitoneal injection following a 12–15 h fasting period. On day 1 of the induction phase, STZ was injected at a concentration of 20 mg/kg body weight. The rats were then fed an unlimited high-fat diet for 2 days, followed by an additional fasting period of 12–15 h. On day 3 of the induction phase, a second STZ injection was administered at a concentration of 30 mg/kg body weight, and the rats were subsequently fed an unlimited high-fat diet for 7 days. The diabetic rats exhibited a mean blood glucose level of 332.90 ± 16.81 mg/dL and were then divided into five groups: the diabetic control group (DM; n = 12), the diabetic group supplemented with 1% inulin (DM + 1% inulin; n = 12), the diabetic group supplemented with 3% inulin (DM + 3% inulin; n = 12), the diabetic group supplemented with 1% IMOs (DM + 1% IMOs; n = 12) and the diabetic group supplemented with 3% IMOs (DM + 3% IMOs; n = 12). a Pictorial representation of the animal experimental design is show in Supplementary Fig. 1. The administration of IMOs and inulin involved substituting cellulose in the diet (Table 1).

### IMOs consumption on body weight and general blood biochemistry

Throughout the study period, weekly measurements were recorded for rat food intake and body weight. Glucose levels were evaluated biweekly by obtaining 0.1 mL of blood from the tail using a venipuncture needle and promptly measuring it with a blood glucose meter (Accu-Check^®^ Performa, Roche Diagnostics, Thailand).

After an 8-week duration, each rat underwent a fasting period lasting approximately 12–16 h and was subsequently euthanized using carbon dioxide, following the established protocol of the laboratory animal facility at the Faculty of Tropical Medicine, Mahidol University (FTM-ACUC 003/2019). Approximately 12 mL of blood was obtained from the vena cava vein and divided into whole blood and plasma. The whole blood was utilized for blood biochemistry analysis using an automated monitor (Cobas^®^ 8800 System, Roche Diagnostics, Switzerland). The plasma was carefully collected and stored at − 20 °C for subsequent analysis. Liver specimens were collected for proteomic analysis, while pancreas specimens were collected for histopathological examination.

### IMOs consumption on inflammatory conditions

We measured tumor necrosis factor-alpha (TNF-α) and C-reactive protein (CRP) levels in plasma using the Enzyme-Linked Immunosorbent Assay (ELISA) method. For TNF-α, we used the Rat Tumor Necrosis Factor α ELISA Kit (Merck KGaA, Germany), and for CRP, we used the Rat C-Reactive Protein ELISA Kit (Merck KGaA, Germany). The ELISA method relies on specific antigen–antibody reactions, followed by absorbance measurements, following the manufacturer’s instructions.

### Intraperitoneal glucose tolerance test (IPGTT)

One week before the end of the experiment, a glucose tolerance test was conducted on all 84 rats. The rats underwent an overnight fasting period lasting approximately 12–16 h before the test. A glucose solution was prepared by dissolving 2 g of glucose per kilogram of body weight in 0.9% sodium chloride. This glucose solution was then injected intraperitoneally into the rats. Blood samples were collected from the tail vein of the rats at various time points: 0, 5, 15, 30, 60, 120, and 180 min after the glucose injection. Blood glucose levels were measured using a blood glucose meter to assess the rats' response to the glucose challenge under inflammatory conditions.

### Histopathological examination

The pancreas was immersed in a 10% buffered formalin solution overnight for fixation, followed by preservation in 70% ethanol for tissue sectioning. Subsequently, the tissue sections were stained with hematoxylin and eosin and examined under a microscope at 40 × magnification for histopathological analysis.

### Proteomic analysis

The rat liver tissues were resuspended in a lysis buffer containing 1% (vol/vol) sodium dodecyl sulfate (SDS), 1% (vol/vol) Triton X-100, and 0.5% (vol/vol) sodium chloride. Ultrasonication (Sonics & Materials, CT, USA) was used to disrupt cellular structures in the homogenate. The protein concentration in the lysate was determined using a QuickStart Bradford protein assay kit (Bio-Rad Laboratories) with bovine serum albumin serving as the protein standard.

Each sample, containing 30 μg of protein was separated by 12% SDS polyacrylamide gel electrophoresis (SDS-PAGE). Following electrophoresis, the protein bands were visualized by staining with Coomassie blue G (Merck KGaA Germany). Gel lanes containing proteins of interest were excised and cut into small pieces. Gel pieces underwent destaining using 50% (vol/vol) acetonitrile in 50 mM ammonium bicarbonate. The proteins in the gel pieces were reduced by incubating with 4 mM dithiothreitol (DTT) at 60 °C for 15 min, followed by alkylation with 250 mM iodoacetamide at room temperature in the dark for 30 min. The alkylation reaction was quenched with 4 mM DTT, and dehydration was achieved by incubation in 100% acetonitrile. Gel pieces were rehydrated with trypsin (10 ng/μL) in 50 mM ammonium bicarbonate and incubated overnight at 37 °C for in-gel digestion of proteins into peptides. The peptides were extracted by adding 100% acetonitrile, collecting the supernatant, and completely drying the peptide mixtures using a SpeedVac concentrator (Tomy, Japan) for subsequent analysis.

The trypsin-digested samples were resuspended in 0.1% (vol/vol) formic acid containing 2% (vol/vol) acetonitrile and analyzed using an UltiMate 3000 Nano LC system (Dionex) coupled with a micrOTOF-Q mass spectrometer (Bruker Daltonics). Data acquisitions were controlled using HyStar software (Bruker Daltonics). MS and MS/MS spectra covered the mass range of m/z 400–2000 and m/z 50–1500, respectively. LC–MS/MS data files were searched against the NCBI database using Mascot v2.4.1 (Matrix Science) for protein identification, with acceptance at a confidence level of 95%. Semi-quantitative analysis of protein abundance relied on the exponentially modified protein abundance index (emPAI), a method widely used for estimating protein abundance from mass spectrometry data. Protein content calculated using emPAI values derived from the dataset and compared resulting data to explore differences across experimental conditions.

### Data analysis

The Shapiro–Wilk test was used to assess the normality of continuous data. An independent samples t-test was used to compare body weight changes between the normal and high-fat diet groups. One-way ANOVA followed by Tukey's post-hoc test was employed to analyze differences in body weight, blood biochemical parameters (lipid profile, glucose), and inflammatory markers across the experimental groups. The statistical significance level was set at 0.05, and results are presented as mean with standard deviation. For the proteomics analysis, differentially expressed proteins were identified based on fold-changes in average abundance between treatment and control groups. Proteins with an abundance ratio (treatment/control) above 1.5 or below 0.67 were considered increased or decreased by treatment. Only differential proteins with at least two biological replications were presented in this manuscript. For gene ontology analysis, p-value ≤ 0.05 was set in the panther software.

## Results

### Production and purification of IMOs

**Fig. 1:**
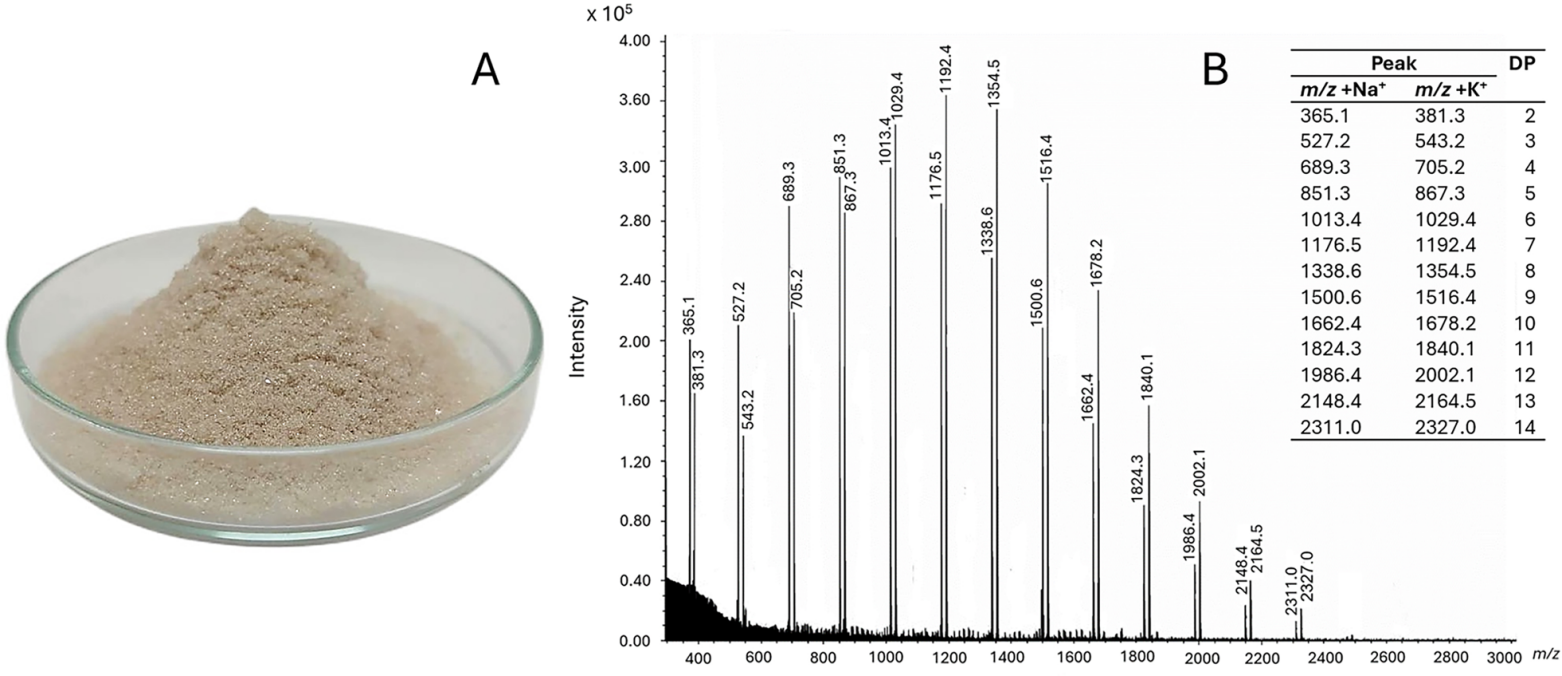
Characteristics of Isomaltooligosaccharides (IMOs). **A** IMOs powder obtained from *B. subtilis* AP-1. **B** MALDI-TOF–MS spectra of IMOs product

The IMOs product was derived from maltose through direct fermentation using *B. subtilis* strain AP-1. After fermentation, a purification process was employed, involving hollow-fiber ultrafiltration with a 10 kDa membrane and activated carbon treatment. This process resulted in a refined product that was subsequently freeze-dried. The freeze-drying method effectively preserved the integrity of the IMOs while removing moisture, resulting in a water-soluble, cream-colored powder (Fig. 1A). The purity of the IMOs product was assessed following the NREL Chemical Analysis and Testing Standard Procedure, revealing a purity of 86.26% (w/w), indicative of a high purity level.

To confirm the composition of the IMOs product, MALDI-TOF–MS analysis was conducted. The MALDI-TOF–MS spectra of the IMOs product (Fig. 1B) revealed a distinct series of peaks corresponding to various degrees of polymerization, ranging from DP2 to DP14. The intensity of the peaks followed the order DP7 > DP6 > DP8 > DP5 > DP9 > DP4 > DP10 > DP3 > DP2 > DP11 > DP12 > DP13 > DP14. Additionally, the MALDI-TOF–MS analysis identified molecular weights corresponding to oligomers of glucose units with DP ranging from 2 to 14. Specifically, the molecular weights were approximately 342 (DP2), 504 (DP3), 666 (DP4), 828 (DP5), 990 (DP6), 1153 (DP7), 1315 (DP8), 1477 (DP9), 1639 (DP10), 1801 (DP11), 1963 (DP12), 2125 (DP13), and 2288 (DP14) Daltons. This analysis confirmed the presence of a diverse array of long-chain IMOs within the product, which was subsequently utilized for further studies.

### Effect of high-fat diet on body weight

**Fig. 2:**
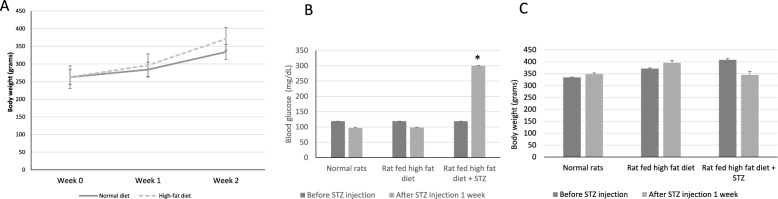
Body weight changes in experimental rat groups. **A** Effect of a high-fat diet on body weight after 2 weeks. **B** Effect of diabetes induction by streptozotocin (STZ) on blood glucose levels. **C** Effect of STZ on body weight

This study assessed the effect of a high-fat diet on the body weight of the experimental rats. Initially, all rat groups had comparable weights (278.00 ± 9.38 g) before the experiment. After a two-week period of consuming a high-fat diet, the group exposed to the high-fat diet (334.71 ± 12.96 g) showed a higher weight compared to the group fed a normal diet (371.25 ± 17.96 g) (Fig. 2A).

### Effect of streptozotocin (STZ) induction on glucose and body weight

Based on the results related to the induction of diabetes by STZ on blood glucose (Fig. 2B) and body weight (Fig. 2C) in rats, before STZ administration, the blood glucose levels were relatively consistent across all rat groups, averaging approximately 108 ± 12.43 mg/dL. However, following the injection of STZ at a dosage of 30 mg/kg of rat body weight, a substantial elevation in glucose levels to approximately 302 ± 12.47 mg/dL was observed. Regarding body weight, following STZ injection, a reduction in body weight of approximately 15.5% was observed.

### Effect of IMOs on body weight and food intake

**Fig. 3:**
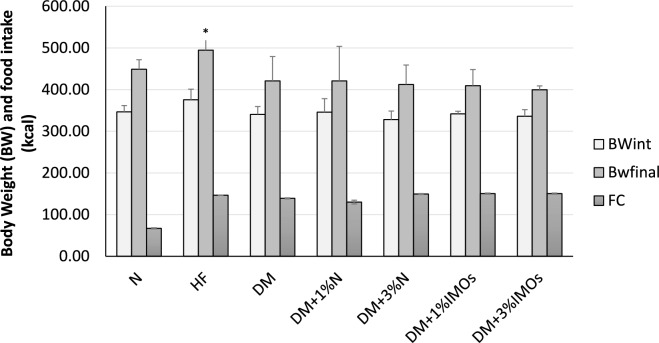
Effect of inulin or IMOs on body weight of diabetic (DM) rats. Prebiotics amount per body weight (g) and food intake (kcal) showed a statistically significant difference (*P* < 0.05) between the rat groups. BWint, Bwfinal and FC are initial body weight, final body weight and food consumption respectively

The weight similarity among all rat groups can be observed in the week preceding the commencement of treatment, as indicated by the white bars (Fig. 3). However, upon administering the treatment along with a high-fat diet for a duration of 4 weeks, the rats fed a high-fat diet exhibited a higher weight compared to the rats receiving a normal diet (gray bars in Fig. 3). Despite consuming a greater number of kilocalories from the high-fat diet compared to the normal diet, all diabetic (DM) rats showed a decrease in body weight. The administration of inulin or IMOs for a duration of 4 weeks had no beneficial impact on the body weight of DM rats (Fig. 3).

### Effects of IMOs on lipid profile and glucose levels

**Fig. 4:**
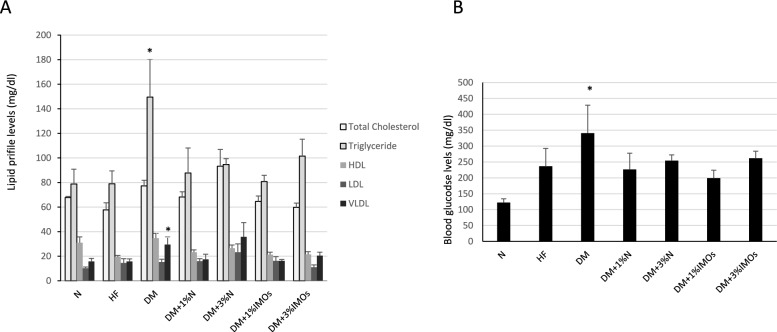
Effects of prebiotics on lipid profile and glucose levels. **A** Prebiotics effects on lipid profile and glucose levels showed significant differences (P < 0.05) between rat groups. **B** Improvement in blood glucose levels across diabetic rat groups, particularly DM + 1%P group. *HDL* High-density lipoprotein, *LDL* Low-density lipoprotein, *VLDL* Very low-density lipoprotein

After a 4-week treatment period (Fig. 4A), the high-fat diet groups displayed a tendency for decreased High Density Lipoprotein (HDL) levels and increased Low Density Lipoprotein (LDL) levels compared to the normal diet (N) groups. Conversely, the DM rats exhibited significantly higher levels of triglycerides and very low-density lipoproteins (VLDL), often referred to as “bad cholesterol,” compared to the other rat groups. Subsequently, upon administering inulin or IMOs to the diabetic rats (DM + 1%N, DM + 3N%, DM + 1%IMOs, DM + 3%IMOs), a noteworthy reduction in triglyceride and VLDL levels was observed compared to the DM group, particularly in the DM + 1%IMOs group.

Regarding blood glucose levels, the N group exhibited normal values, while the high-fat diet group displayed elevated glucose levels. Notably, the DM group of rats exhibited the highest blood glucose levels. However, following the administration of inulin or IMOs, an improvement in blood sugar levels was observed across all groups of diabetic rats, particularly in the DM + 1%IMOs group (Fig. 4B).

### Effects of IMOs on liver and kidney indicators

**Fig. 5:**
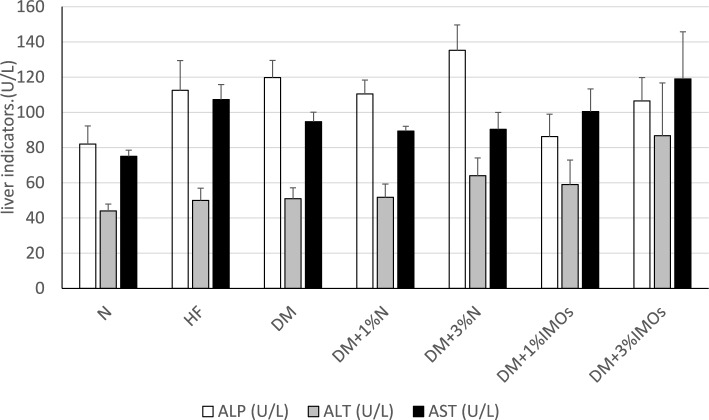
Effects of prebiotics on liver function markers

The rats in the group N, which were fed a normal diet, exhibited the lowest levels of liver alkaline phosphatase (ALP), alanine transaminase (ALT), and aspartate transaminase (AST). In contrast, the levels of these liver indices were higher in the high-fat diet and DM groups compared to those fed normal diets. However, the administration of inulin or IMOs for a duration of 4 weeks did not result in any statistically significant effects on the levels of ALP, AST, and ALT (Fig. 5). Similarly, the renal indices, blood urea nitrogen, and creatinine, exhibited values within the range of 13.6–17.4 mg/dL and 0.24–0.30 mg/dL, respectively, without significant statistical differences. Although not statistically significant, the experimental results suggest that the DM + 3% N group displayed the highest ALP value. This observation may indicate that a 3% inulin concentration has an effect on the liver, leading to an increase in ALP levels. Additionally, the DM + 3% IMOs group exhibited the highest values of ALT and AST. Hence, it is plausible that higher doses of the supplement may have an impact on these liver indices.

### Effects of IMOs on the immune system and inflammation

The analysis of experimental results revealed no statistically significant differences in total white blood cell counts across all rat groups (Table 2). Groups N and HF exhibited comparable levels of total white blood cells. However, the DM group displayed a tendency toward decreased white blood cell counts, suggesting a compromised immune system. Treatment with inulin (1% and 3% groups) led to elevated white blood cell counts, surpassing those observed in group N, indicative of a heightened immune status. Notably, the IMOs-treated groups (DM + 1%IMOs, DM + 3%IMOs) demonstrated total white blood cell counts similar to the group N, implying a favorable impact on the immune system.

**Table 2:** Effect of IMOs on white blood cell count

	N	HF	DM	DM + 1%N	DM + 3%N	DM + 1%IMOs	DM + 3%IMOs
Total WBC	13.17 ± 2.87	13.17 ± 0.65	11.89 ± 1.96	16.27 ± 1.76	15.81 ± 1.32	13.65 ± 0.83	13.23 ± 0.64
% Neutrophil	26.17 ± 11.79	22.93 ± 12.64	16.53 ± 3.02	16.63 ± 3.04	19.70 ± 4.17	14.17 ± 1.71	15.23 ± 0.79
% Lymphocyte	61.47 ± 10.38	70.05 ± 8.57	72.57 ± 1.14	79.13 ± 3.52	70.98 ± 3.86	76.53 ± 3.18	75.55 ± 3.08
% Monocyte	7.03 ± 1.13	3.98 ± 0.84	6.50 ± 0.83	3.03 ± 0.92	6.35 ± 1.20	5.25 ± 2.10	5.83 ± 1.93
% Eosinophil	1.60 ± 0.31	1.45 ± 0.75	1.53 ± 0.44	1.15 ± 0.16	1.80 ± 0.26	1.90 ± 0.24	2.28 ± 0.17
% Basophil	3.73 ± 2.64	1.43 ± 0.72	2.87 ± 2.27	1.15 ± 0.41	2.93 ± 0.90	2.95 ± 1.58	2.05 ± 1.03

*N* Rat groups receiving normal diets, *HF* Rat groups receiving high-fat diets, *DM* Rat groups induced with diabetes and receiving high-fat diets, *DM* + *1% inulin* Diabetic-induced rat group administered with 1% inulin and high-fat diets, *DM* + *3% inulin* Diabetic-induced rat group administered with 3% inulin and high-fat diets, *DM* + *1% IMOs* Diabetic-induced rat group administered with 1% IMOs and high-fat diets, *DM* + *3% IMOs* Diabetic-induced rat group administered with 3% IMOs and high-fat diets

**Fig. 6:**
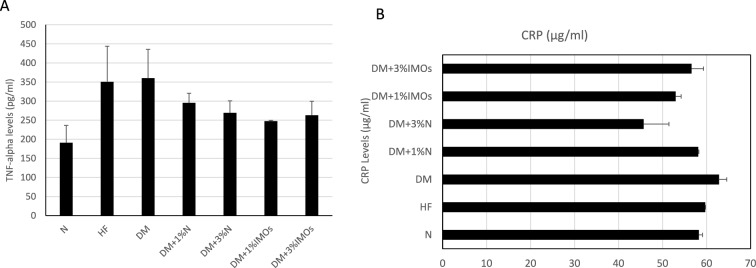
Effects of prebiotics on inflammatory markers. **A** Prebiotics effects on tumor necrosis factor-alpha (TNF-α) levels. **B** Prebiotics effects on C-reactive protein (CRP) levels

Figure 6A illustrates a trend where the high-fat diet and DM groups exhibit higher TNF-alpha levels compared to the N group. However, administration of inulin or IMOs shows a trend toward reducing TNF-alpha levels, aligning with the observed direction of white blood cell count changes. CRP levels were higher in the DM group compared to the high-fat diet group (Fig. 6B). Additionally, the high-fat diet group shows a tendency for elevated CRP levels compared to the N group. Nonetheless, supplementation with inulin or IMOs demonstrates a decreasing trend in CRP levels relative to the high-fat diet and DM groups, which aligns with the direction of TNF-alpha levels.

### Glucose tolerance test (intraperitoneal glucose tolerance test)

**Fig. 7:**
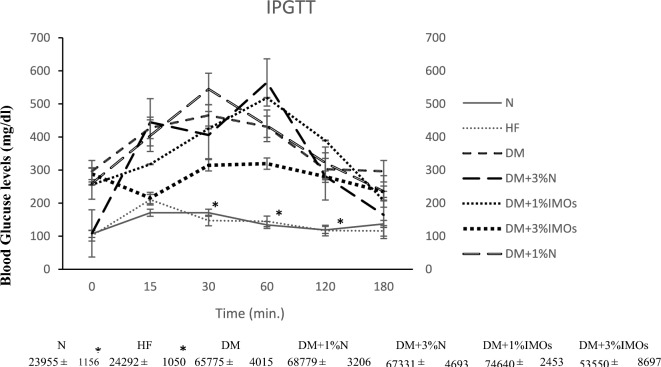
Effects of prebiotics on glucose tolerance assessed by intraperitoneal glucose tolerance test (IPGTT). *Indicates statistically significant difference (*P* < 0.05)

**Fig. 8:**
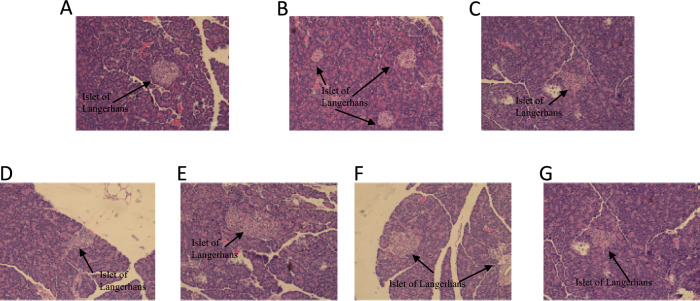
(**A**) Normal control (N) (**B**) High-fat diet (HF) (**C**) Diabetic (DM) (**D**) DM + 1% inulin (**E**) DM + 3% inulin (**F**) DM + 1% IMOs (**G**) DM + 3% IMOs

The Glucose Tolerance Test (IPGTT) is utilized to assess pancreatic β-cell function in glucose transportation into cells. IPGTT results were analyzed by the Area Under the Curve (AUC). Normal rats maintain normal glucose levels within 2 h. The AUC did not show significant differences between the N and high-fat diet groups, indicating normal glucose uptake by cells (Fig. 8). However, the DM group exhibited impaired glucose utilization associated with diabetes, demonstrating a significantly increased AUC compared to other groups at a significance level of 0.05 (*P* < 0.05). The AUC of the DM group was similar across all treatment groups, including those treated with 1% and 3% inulin and IMOs. Although the 3% IMOs group displayed a tendency toward improved glucose tolerance compared to the DM group, the difference was not statistically significant (Fig. 7).

### Histological examination of pancreas pathology

Microscopic examination of pancreas sections showed no noticeable differences between the N and high-fat diet groups. Both groups exhibited normal beta cell appearance and quantity. However, comparison with the DM group revealed abnormalities in beta cells, characterized by a reduced cell count and diminished size. Importantly, the pancreatic pathology observed in the DM group resembled that of all treatment groups, indicating that the administration of 1 and 3% inulin and IMOs did not affect pancreatic cell morphology (Fig. 8). The observed reduction in glucose levels was consistent with the pathological findings, indicating that IMOs had no impact on pancreatic cell function.

### Proteomic analysis

Although no statistically significant differences were observed, diabetic and hyperlipidemic rats treated with 1% and 3% IMOs exhibited lower blood sugar levels and triglycerides compared to those treated with inulin, indicating potential beneficial effects of IMOs on blood sugar and lipid metabolism. Additionally, they showed adjustments in white blood cell levels and CRP levels that were closer to those of the normal group. Consequently, we proceeded to separate proteins from the livers of untreated diabetic and hyperlipidemic rats and those treated with 1% IMOs by SDS-PAGE, followed by staining the resulting gels with Coomassie blue. Subsequently, each lane was cut into small pieces, in-gel digestion was performed, and the peptide extracts were analyzed by MS. A total of 873 proteins were identified in the livers. The hepatic protein profiles of the untreated diabetic and hyperlipidemic rats and those treated with 1% IMOs were compared, revealing 210 upregulated proteins and 278 downregulated proteins in the IMOs-treated group. Gene ontology (GO) classification of the differentially expressed proteins demonstrated that cellular processes (27.9%), catalytic activity (38.5%), and cell part (21.9%) were the major classes under the biological process, molecular function, and cellular component terms, respectively (Table 3). The top 20 most upregulated and downregulated proteins are presented in Tables 4 and 5, respectively.

**Table 3:** Gene ontology (GO) classification of the differentially expressed proteins

No.	Gene ontology	Protein	Percent (%)
Molecular function term			
1	Translation regulator activity (GO:0045182)	2	0.60
2	Transcription regulator activity (GO:0140110)	14	4.30
3	Molecular transducer activity (GO:0060089)	14	4.30
4	Binding (GO:0005488)	116	35.70
5	Structural molecule activity (GO:0005198)	24	7.40
6	Molecular function regulator (GO:0098772)	11	3.40
7	Catalytic activity (GO:0003824)	125	38.50
8	Transporter activity (GO:0005215)	19	5.80
Biological process term			
1	Developmental process (GO:0032502)	22	3.00
2	Multicellular organismal process (GO:0032501)	28	3.80
3	Cellular process (GO:0009987)	204	27.90
4	Reproduction (GO:0000003)	4	0.50
5	Cell population proliferation (GO:0008283)	2	0.30
6	Localization (GO:0051179)	61	8.30
7	Reproductive process (GO:0022414)	4	0.50
8	Multi-organism process (GO:0051704)	6	0.80
9	Biological adhesion (GO:0022610)	5	0.70
10	Immune system process (GO:0002376)	6	0.80
11	Cellular component organization or biogenesis (GO:0071840)	68	9.30
12	Biological regulation (GO:0065007)	86	11.80
13	Signaling (GO:0023052)	29	4.00
14	Metabolic process (GO:0008152)	136	18.60
15	Response to stimulus (GO:0050896)	56	7.70
16	Biomineralization (GO:0110148)	1	0.10
17	Biological phase (GO:0044848)	1	0.10
18	Behavior (GO:0007610)	1	0.10
19	Rhythmic process (GO:0048511)	1	0.10
20	Locomotion (GO:0040011)	10	1.40
Cellular component term			
1	Synapse part (GO:0044456)	12	1.30
2	Membrane part (GO:0044425)	47	4.90
3	Membrane (GO:0016020)	79	8.30
4	Synapse (GO:0045202)	12	1.30
5	Organelle part (GO:0044422)	82	8.60
6	Extracellular region part (GO:0044421)	20	2.10
7	Cell junction (GO:0030054)	3	0.30
8	Membrane-enclosed lumen (GO:0031974)	17	1.80
9	Protein-containing complex (GO:0032991)	76	8.00
10	Supramolecular complex (GO:0099080)	10	1.10
11	Extracellular region (GO:0005576)	20	2.10
12	Cell (GO:0005623)	208	21.90
13	Cell part (GO:0044464)	208	21.90
14	Organelle (GO:0043226)	156	16.40

**Table 4:** The top 20 most upregulated proteins

No.	Accession no.	Protein	Score	M.W.	No. of peptide	%Cov	pI	Fold change
1	KHK_RAT	Ketohexokinase OS=Rattus norvegicus GN=Khk PE=1 SV=1	281	32729	6	26.5	6.24	5.65
2	TKT_RAT	Transketolase OS=Rattus norvegicus GN=Tkt PE=1 SV=1	924	67601	23	45.4	7.23	4.90
3	DHSO_RAT	Sorbitol dehydrogenase OS=Rattus norvegicus GN=Sord PE=1 SV=4	137	38210	8	28.6	7.14	4.44
4	PNPH_RAT	Purine nucleoside phosphorylase OS=Rattus norvegicus GN=Np PE=1 SV=1	264	32281	10	44.3	6.46	4.41
5	CX6A1_RAT	Cytochrome c oxidase subunit 6A1, mitochondrial OS=Rattus norvegicus GN=Cox6a1 PE=1 SV=2	65	12293	2	33.3	9.3	3.89
6	RGN_RAT	Regucalcin OS=Rattus norvegicus GN=Rgn PE=1 SV=3	1672	33368	22	72.6	5.27	3.38
7	MGST1_RAT	Microsomal glutathione S-transferase 1 OS=Rattus norvegicus GN=Mgst1 PE=1 SV=3	223	17460	6	38.1	9.63	3.32
8	RS19_RAT	40S ribosomal protein S19 OS=Rattus norvegicus GN=Rps19 PE=2 SV=3	86	16076	5	33.8	10.41	2.90
9	UD17_RAT	UDP-glucuronosyltransferase 1-7 OS=Rattus norvegicus GN=Ugt1 PE=2 SV=1	292	59589	7	16.6	8.6	2.83
10	APOA1_RAT	Apolipoprotein A-I OS=Rattus norvegicus GN=Apoa1 PE=1 SV=2	233	30043	8	42.9	5.52	2.73
11	H2A1_RAT	Histone H2A type 1 OS=Rattus norvegicus PE=1 SV=2	667	14069	8	57.7	10.9	2.57
12	MDHM_RAT	Malate dehydrogenase, mitochondrial OS=Rattus norvegicus GN=Mdh2 PE=1 SV=2	2326	35661	27	68.9	8.93	2.45
13	H2B1_RAT	Histone H2B type 1 OS=Rattus norvegicus PE=1 SV=2	738	13982	8	48	10.36	2.34
14	COX5A_RAT	Cytochrome c oxidase subunit 5A, mitochondrial OS=Rattus norvegicus GN=Cox5a PE=1 SV=1	61	16119	5	30.8	6.08	2.24
15	DEST_RAT	Destrin OS=Rattus norvegicus GN=Dstn PE=1 SV=3	32	18522	6	48.5	8.19	2.22
16	REEP6_RAT	Receptor expression-enhancing protein 6 OS=Rattus norvegicus GN=Reep6 PE=2 SV=1	80	23298	4	23.7	8.39	2.21
17	RS4X_RAT	40S ribosomal protein S4, X isoform OS=Rattus norvegicus GN=Rps4x PE=2 SV=2	63	29579	7	26.6	10.16	2.18
18	K2C7_RAT	Keratin, type II cytoskeletal 7 OS=Rattus norvegicus GN=Krt7 PE=2 SV=1	60	50678	8	25.8	5.67	2.17
19	ADT1_RAT	ADP/ATP translocase 1 OS=Rattus norvegicus GN=Slc25a4 PE=1 SV=3	93	32968	14	32.9	9.81	2.10
20	SPRE_RAT	Sepiapterin reductase OS=Rattus norvegicus GN=Spr PE=1 SV=1	101	28110	4	21.4	5.55	2.08

**Table 5:** The top 20 most downregulated proteins

No.	Accession no.	Protein	Score	M.W.	No. of peptide	%Cov	pI	Fold change
1	CP2E1_RAT	Cytochrome P450 2E1 OS=Rattus norvegicus GN=Cyp2e1 PE=1 SV=4	135	56591	12	28.4	8.6	6.67
2	RS11_RAT	40S ribosomal protein S11 OS=Rattus norvegicus GN=Rps11 PE=1 SV=3	240	18419	8	50	10.31	4.78
3	ACSM5_RAT	Acyl-coenzyme A synthetase ACSM5, mitochondrial OS=Rattus norvegicus GN=Acsm5 PE=2 SV=1	200	64582	11	29.2	8.12	3.80
4	RL11_RAT	60S ribosomal protein L11 OS=Rattus norvegicus GN=Rpl11 PE=1 SV=2	171	20240	7	32.6	9.64	3.65
5	ASSY_RAT	Argininosuccinate synthase OS=Rattus norvegicus GN=Ass1 PE=2 SV=1	1883	46467	21	42.5	7.63	3.43
6	CP2C6_RAT	Cytochrome P450 2C6 OS=Rattus norvegicus GN=Cyp2c6 PE=2 SV=2	33	55967	13	24.9	7.21	3.17
7	RL24_RAT	60S ribosomal protein L24 OS=Rattus norvegicus GN=Rpl24 PE=1 SV=1	280	17768	5	37.6	11.26	2.99
8	FABPL_RAT	Fatty acid-binding protein, liver OS=Rattus norvegicus GN=Fabp1 PE=1 SV=1	1855	14263	14	89	7.79	2.87
9	SPA3K_RAT	Serine protease inhibitor A3K OS=Rattus norvegicus GN=Serpina3k PE=1 SV=3	86	46532	8	30.5	5.31	2.79
10	ACON_RAT	Aconitate hydratase, mitochondrial OS=Rattus norvegicus GN=Aco2 PE=1 SV=2	284	85380	11	20.4	7.87	2.75
11	SPEB_RAT	Agmatinase, mitochondrial OS=Rattus norvegicus GN=Agmat PE=2 SV=1	179	37963	3	17.8	6.71	2.72
12	RS7_RAT	40S ribosomal protein S7 OS=Rattus norvegicus GN=Rps7 PE=1 SV=1	154	22113	8	40.7	10.09	2.69
13	PARK7_RAT	Protein DJ-1 OS=Rattus norvegicus GN=Park7 PE=1 SV=1	247	19961	8	59.8	6.32	2.69
14	FTHFD_RAT	10-formyltetrahydrofolate dehydrogenase OS=Rattus norvegicus GN=Aldh1l1 PE=1 SV=2	83	99064	17	25.2	5.79	2.67
15	ARLY_RAT	Argininosuccinate lyase OS=Rattus norvegicus GN=Asl PE=2 SV=1	254	51517	15	40.3	5.99	2.67
16	PPIB_RAT	Peptidyl-prolyl cis-trans isomerase B OS=Rattus norvegicus GN=Ppib PE=2 SV=3	300	23788	10	43.1	9.5	2.57
17	H4_RAT	Histone H4 OS=Rattus norvegicus GN=Hist1h4b PE=1 SV=2	250	11360	4	42.7	11.36	2.45
18	ATP5H_RAT	ATP synthase subunit d, mitochondrial OS=Rattus norvegicus GN=Atp5h PE=1 SV=3	1080	18752	9	64.6	6.17	2.43
19	M2GD_RAT	Dimethylglycine dehydrogenase, mitochondrial OS=Rattus norvegicus GN=Dmgdh PE=1 SV=1	652	95987	19	27.9	6.91	2.39
20	TRPV1_RAT	Transient receptor potential cation channel subfamily V member 1 OS=Rattus norvegicus GN=Trpv1 PE=1 SV=1	26	94887	7	10.4	7.96	2.33

Notable findings include the upregulation of Ketohexokinase, Transketolase, Sorbitol dehydrogenase, and Purine nucleoside phosphorylase, with fold differences of 5.65, 4.90, 4.44, and 4.00, respectively. Conversely, Cytochrome P450 2E1, 40S ribosomal protein S11, Acyl-coenzyme A synthetase ACSM5, and 60S ribosomal protein L11 exhibited downregulation, with fold differences of 6.67, 4.78, 3.80, and 3.65, respectively.

**Fig. 9:**
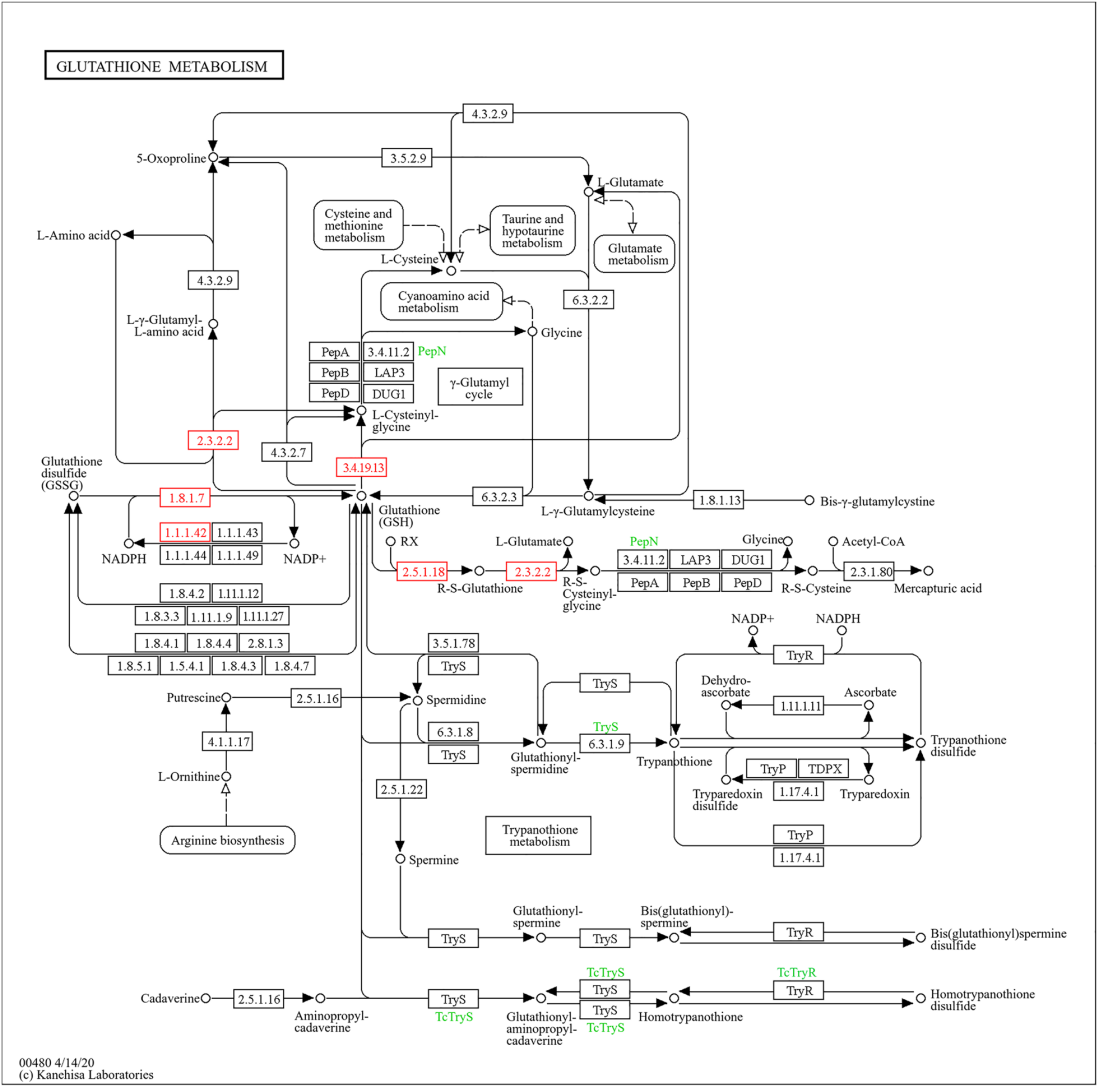
Pathway analysis showing upregulation of glutathione metabolism in response to IMOs treatment, highlighting key enzymes involved in cellular antioxidant defense

**Fig. 10:**
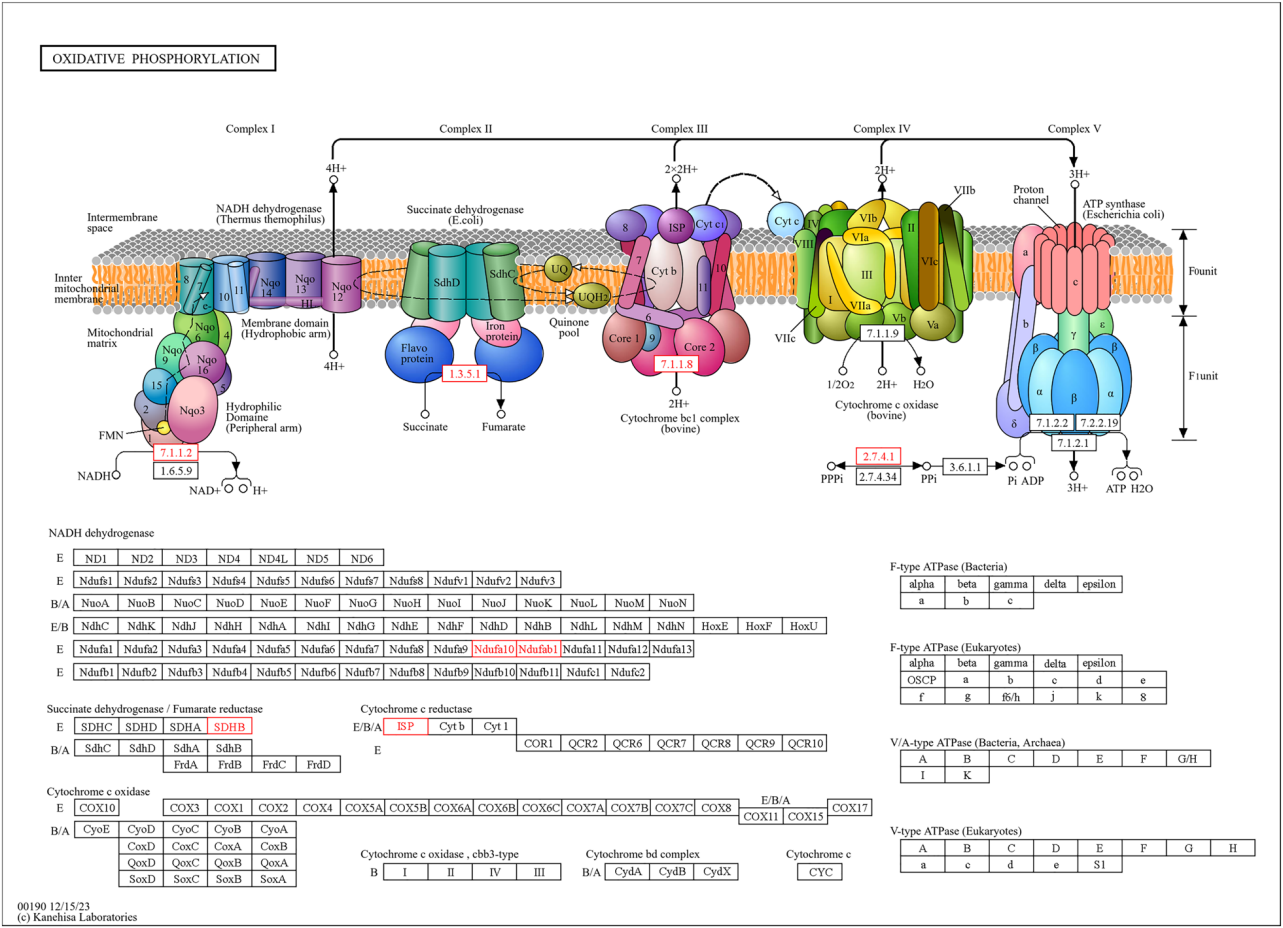
Pathway analysis illustrating upregulation of oxidative phosphorylation in response to IMOs treatment, indicating increased energy demand or metabolic activity

**Fig. 11:**
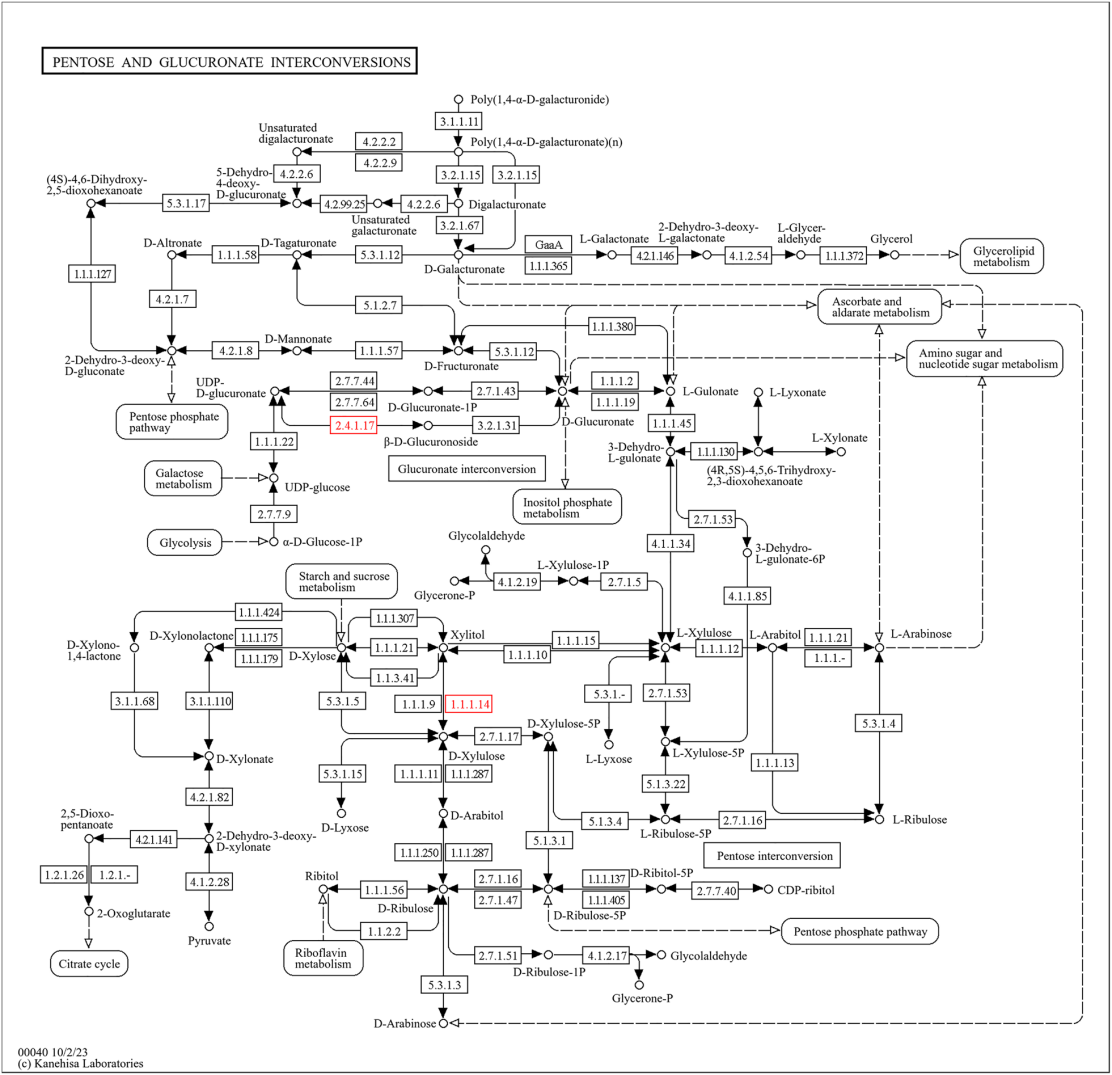
Pathway analysis demonstrating upregulation of pentose and glucuronate interconversions following IMOs treatment, suggesting altered carbohydrate and nucleotide metabolism

Furthermore, the differentially expressed proteins were subjected to pathway analysis using Blast2Go software, revealing upregulation of glutathione metabolism, oxidative phosphorylation, and pentose and glucuronate interconversion pathways. The glutathione metabolism pathway showed upregulation of enzymes such as gamma-glutamyltransferase [EC:2.3.2.2], glutathione reductase [EC:1.8.1.7], isocitrate dehydrogenase [EC:1.1.1.42], glutathione gamma-glutamate hydrolase [EC 3.4.19.13], and glutathione S-transferase [EC:2.5.1.18] (Fig. 9). Similarly, the oxidative phosphorylation pathway displayed upregulation of NADH dehydrogenase (ubiquinone) 1 alpha subcomplex subunit 10, NADH dehydrogenase (ubiquinone) 1 alpha/beta subcomplex 1, succinate dehydrogenase, and cytochrome reductase (Fig. 10). The pentose and glucuronate interconversion pathway exhibited upregulation of glucuronosyltransferase [EC:2.4.1.17] and L-iditol 2-dehydrogenase [EC:1.1.1.14] (Fig. 11).

**Fig. 12:**
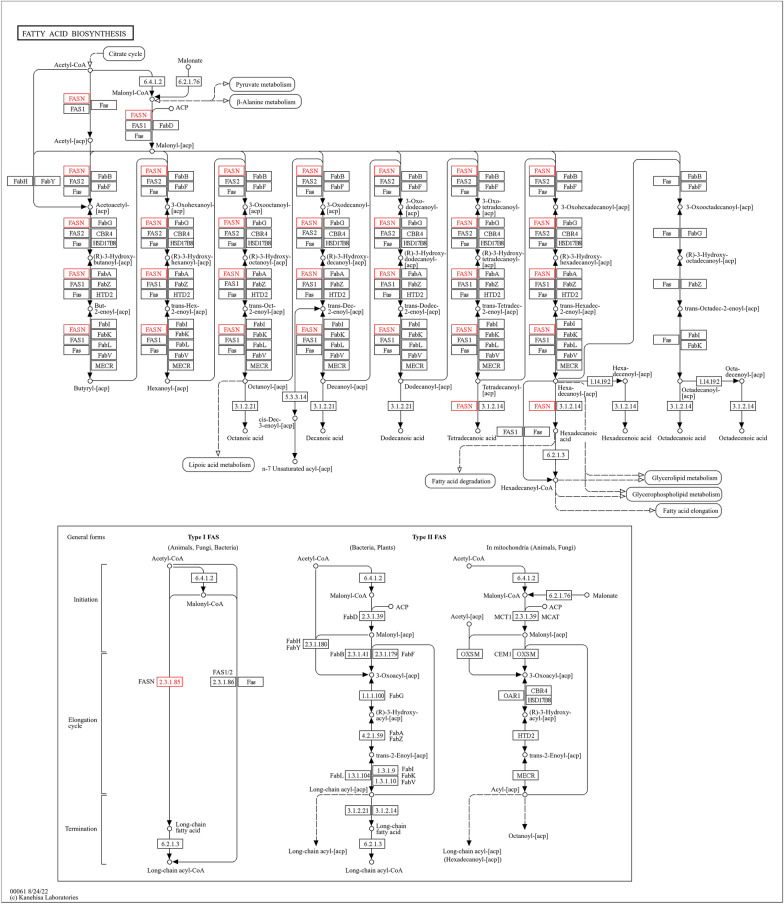
Pathway analysis highlighting downregulation of fatty acid biosynthesis in response to IMOs treatment, a favorable shift for diabetes and hyperlipidemia

**Fig. 13:**
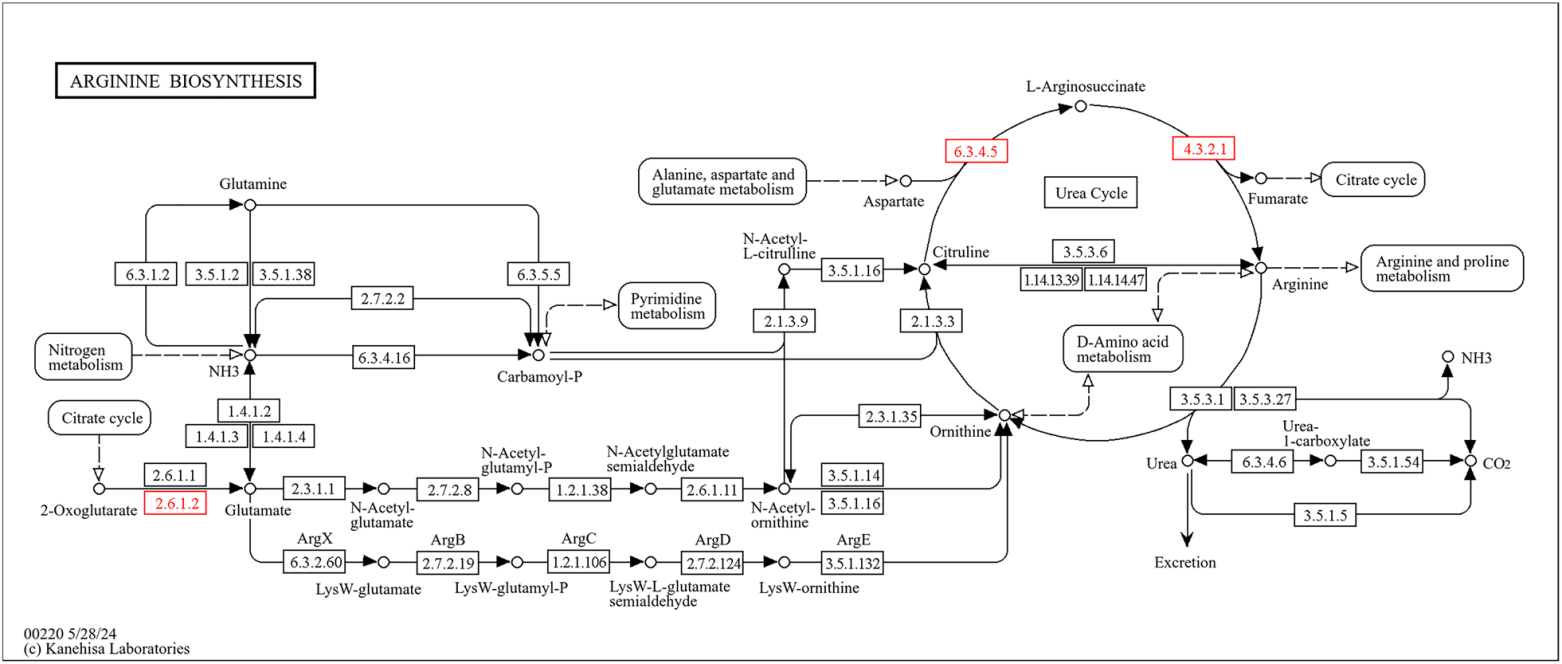
Pathway analysis of arginine biosynthesis illustrating downregulation of enzymes in this pathway in response to IMOs treatment

**Fig. 14:**
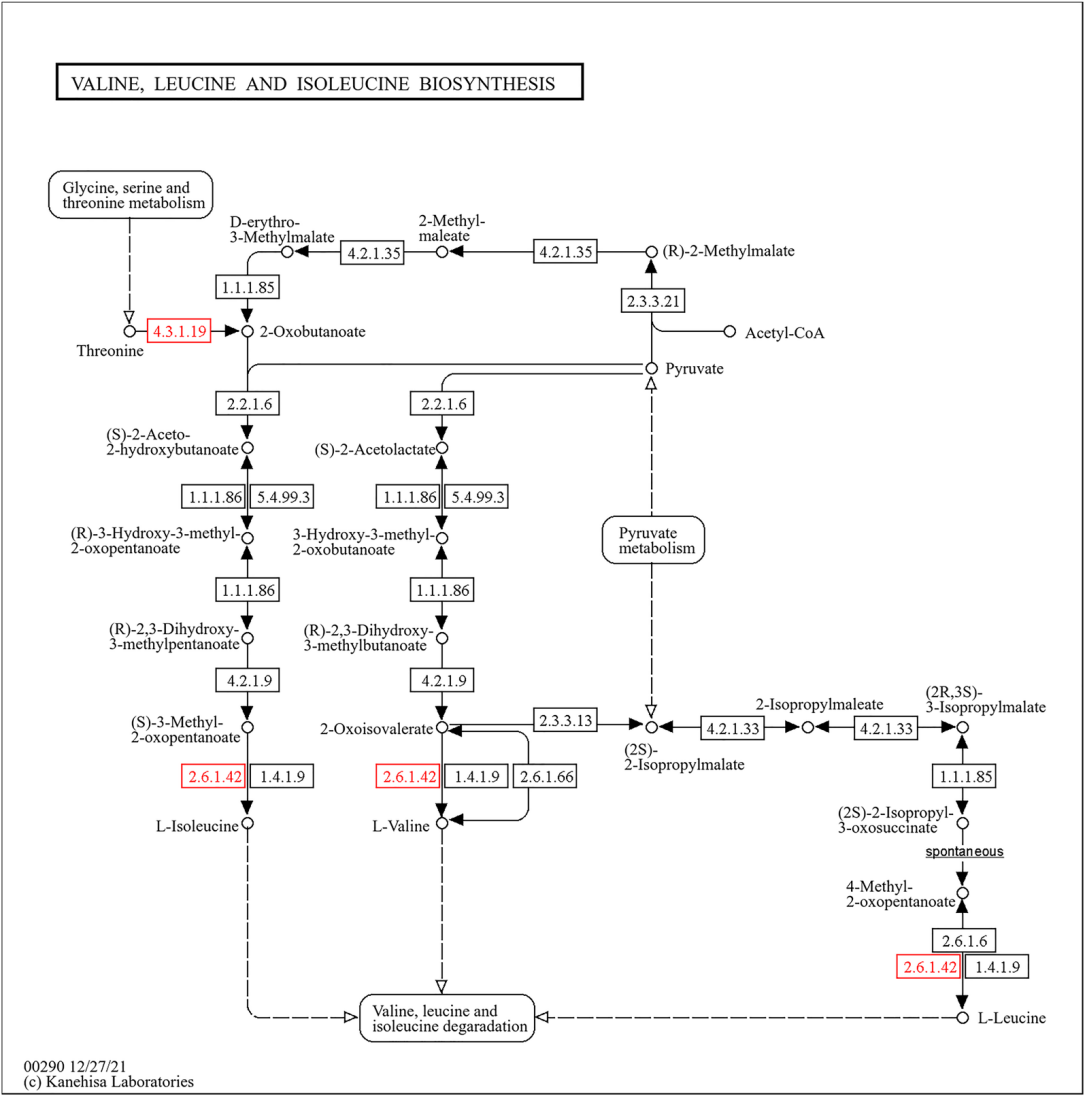
Pathway analysis showing downregulation of valine, leucine, and isoleucine biosynthesis in response to IMOs treatment, indicating reduced amino acid metabolism associated with improved glycemic control

Conversely, pathways involved in fatty acid biosynthesis, arginine biosynthesis, and valine, leucine, and isoleucine biosynthesis exhibited downregulation in response to IMOs treatment. Specifically, key enzymes such as fatty acid synthase [EC:2.3.1.85] and long-chain acyl-CoA synthetase [EC:6.2.1.3] were downregulated in the fatty acid biosynthesis pathway (Fig. 12). Similarly, enzymes involved in arginine biosynthesis, including alanine transaminase [EC:2.6.1.2], argininosuccinate synthase [EC:6.3.4.5], and argininosuccinate lyase [EC:4.3.2.1], showed decreased expression levels (Fig. 13). Likewise, pathways related to valine, leucine, and isoleucine biosynthesis demonstrated downregulation of enzymes such as L-serine/L-threonine ammonia-lyase [EC:4.3.1.17, 4.3.1.19] and branched-chain amino acid aminotransferase [EC:2.6.1.42] (Fig. 14).

## Discussion

Individuals with diabetes commonly experience complications associated with elevated blood glucose and lipid levels, significantly increasing the risk of cardiovascular disease [22]. To simulate the characteristics of Type 2 diabetes mellitus (T2DM) with hyperlipidemia, this study employed a combination of a high-fat diet and low-dose STZ injections to induce minimal pancreatic damage. The rats with induced DM utilized in this study demonstrated evident pathophysiological alterations resembling those observed in patients with Type 2 diabetes. These changes included elevated glucose levels and weight loss. Notably, throughout the 4-week treatment period, all high-fat diet groups consumed an equivalent amount of food; however, all DM groups exhibited lower weight gain compared to high-fat diet groups, consistent with the symptoms observed in individuals with diabetes. This weight loss in the DM groups may be attributed to the body's inability to utilize glucose as an energy source, leading to a state of apparent starvation and subsequent utilization of muscle protein for energy instead [23].

In our experiment, DM rats fed a high-fat diet exhibited the highest triglyceride levels. Conversely, treatment with 1% inulin and IMOs, as well as 3% inulin, resulted in reduced triglyceride levels. Inulin has been extensively demonstrated to lower lipid levels in both human [24] and rat [25–27] subjects, potentially through two mechanisms. First, it diminishes cholesterol absorption by intestinal epithelial cells. Second, it is metabolized by intestinal microflora into short-chain fatty acids (SCFAs), such as acetate, butyrate, and propionate, thereby inhibiting cholesterol synthesis and fatty acid production. Although IMOs have not the exact structure to native inulin, they may exert similar mechanisms as inulin in modulating lipid levels by being water-soluble and viscous compounds, both inulin and IMOs could impede cholesterol absorption within the small intestine. Hence, IMOs may undergo microbial fermentation in the gut, yielding SCFAs that can suppress cholesterol and lipid biosynthesis. The precise mechanism underlying the lipid-reducing effects of inulin remains unclear. However, it is postulated that inulin involves the regulation of gene expression by hormones associated with lipid synthesis, such as glucagon and insulin [28]. Consequently, the presence of stomach and intestinal discomfort may contribute to stress levels in the body and potentially impact hormone secretion.

When examining glucose values in the treated diabetic rats, it was observed that blood glucose levels improved across all groups, with the DM + 1%IMOs group displaying the most notable improvement. Additionally, IMOs-treated diabetic rats (DM + 1%IMOs, DM + 3%IMOs) exhibited improved IPGTT comparable to the N group, suggesting a positive impact on moderating hyperglycemia.

These findings align with meta-analyses and recent studies demonstrating the beneficial effects of inulin in reducing blood sugar levels in individuals with Type 2 diabetes [29]. The proposed mechanism involves the elevation of serum glucagon-like peptide-1 (GLP-1) levels, which subsequently reduce serum interleukin-6 (IL-6) levels and downregulate IL-6 expression in white adipose tissue, serving as an inflammatory marker in mice with diabetes [27, 29–31]. However, further investigations are imperative to expand our understanding of the GLP-1 mechanism and the precise mode of action of our IMOs.

Short-chain fatty acids (SCFAs), notably acetate, propionate, and butyrate, are known to serve as crucial regulators of metabolism and play a role in enhancing immune function. These SCFAs are produced by anaerobic fermentation of indigestible dietary fibers like IMOs and inulin by the gut microbiota [31]. While we did not directly measure SCFA levels in this study, previous research has shown that butyrate supplementation can confer protection against insulin resistance and obesity in rat models [27], and a recent clinical trial demonstrated reduced blood glucose levels and waist-to-hip ratio in patients with T2DM following butyrate supplementation [32]. Our results with the novel IMOs exhibited comparable effects to inulin in modulating levels of white blood cells and C-reactive protein (CRP) to approximate those of the normal group of rats, potentially mediated by increased SCFA production.

The use of IMOs at a 1% dose demonstrated superiority compared to 1% inulin in achieving the observed outcomes, including the reduction of CRP levels. CRP serves as a biomarker for assessing inflammation, monitoring disease progression, and predicting the onset of specific conditions, notably cardiovascular disease and diabetes [33]. Similar anti-inflammatory effects of inulin have been reported previously [32], further supporting the potential therapeutic benefits of IMOs. Consequently, further investigations are warranted to elucidate the precise mechanisms of action of our IMOs.

Inulin is widely accepted as a food ingredient without significant dietary restrictions in most countries. Previous studies have documented that inulin is commonly consumed in the United States and Europe, with an average daily intake of approximately 10 g. However, excessive consumption may lead to bloating in some individuals, and the increased osmotic pressure in the gastrointestinal tract can result in feelings of intestinal discomfort [34]. In this study, concentrations of 1% inulin or 1% IMOs and 3% inulin or 3% IMOs were utilized, and their equivalence to human consumption was estimated using the Km factor [35]. For a 60 kg adult, these concentrations corresponded to approximately 8 g and 20 g, respectively, with 3% inulin considered a relatively high concentration. This observation provides a potential explanation for the superior performance of the 1% dose compared to the 3% dose in our study.

Although no statistical significance was observed, the DM + 3%N group exhibited the highest ALP value, whereas the DM + 3%P group had the highest ALT and AST values. The elevated ALP levels suggest that supplementation with 3% inulin and 3% IMOs is equivalent to the addition of inulin or IMOs at a higher amount, approximately 20 g more for a 60 kg adult, which is considered a relatively high dosage as typically only 5 g is added [34]. This may potentially impact liver function. The experimental findings were consistent with blood lipid values observed in the 3% inulin and 3% IMOs groups, indicating the potential unfavorable effects at higher dose.

Diabetic rats treated with 1% and 3% inulin demonstrated reductions in blood glucose and triglyceride levels. Similarly, diabetic rats treated with 1% and 3% IMOs exhibited effects similar to inulin, aiding in the normalization of white blood cell levels and CRP, comparable to the normal group of rats. These findings are in line with previous studies demonstrating the beneficial effects of inulin and its derivatives on glycemic control and lipid metabolism [32]. These results provide valuable insights for further exploration into the mechanisms and benefits of this IMOs, potentially leading to the development of novel products in the future. Furthermore, it may offer an alternative for individuals experiencing difficulties in sugar and fat metabolism, reducing reliance on imported medications or supplements.

Glutathione metabolism plays a vital role in cellular defense against oxidative stress and detoxification [37]. The upregulation of key enzymes such as gamma-glutamyltransferase, glutathione reductase, glutathione gamma-glutamate hydrolase, and glutathione S-transferase in response to IMOs treatment suggests an enhanced antioxidant response and detoxification capacity in rat livers, which is particularly relevant for individuals with diabetes and hyperlipidemia. Both conditions are associated with increased oxidative stress and impaired antioxidant defenses. Continued high blood sugar levels and changes in lipid levels in diabetes lead to the production of reactive oxygen species, resulting in oxidative damage and inflammation [3]. The observed upregulation of glutathione metabolism enzymes may represent a compensatory mechanism to counteract oxidative stress in the liver of diabetic and hyperlipidemic individuals, although further research is needed to fully understand its implications and therapeutic potential.

Oxidative phosphorylation, the primary mechanism for ATP production, shows upregulation in response to IMOs treatment, indicating intensified energy demand or metabolic activity. This metabolic response has implications for hyperglycemia and hyperlipidemia, as both conditions involve metabolic dysregulation and altered energy metabolism. In diabetes, insulin resistance disrupts glucose utilization, leading to increased reliance on alternative energy sources such as fatty acids, which are metabolized through oxidative phosphorylation [37]. Additionally, hyperlipidemia, characterized by elevated lipid levels, can impair oxidative phosphorylation [38]. The observed upregulation in response to IMOs treatment may represent a compensatory mechanism to meet increased energy demands associated with these metabolic disturbances, suggesting potential therapeutic benefits.

The pentose and glucuronate interconversion pathway are crucial for carbohydrate metabolism and nucleotide synthesis, exhibiting upregulation in response to IMOs treatment, indicating potential alterations in these processes. This pathway plays a crucial role in various biosynthesis pathways such as lipogenesis and gluconeogenesis [39]. Therefore, the observed upregulation holds therapeutic promise for both diabetes and hyperlipidemia. In diabetes, enhanced glucose utilization and metabolism may regulate blood glucose levels and mitigate cell damage. Similarly, involvement in lipid metabolism and glycosaminoglycan synthesis suggests potential benefits for mitigating vascular damage and reducing cardiovascular risk in hyperlipidemia [40]. These findings highlight the potential of IMOs treatment to enhance glycemic control and lipid metabolism, offering therapeutic benefits for associated complications.

The downregulation of fatty acid and amino acid biosynthesis pathways in response to IMOs treatment indicates a favorable metabolic shift for diabetes and hyperlipidemia. In diabetes, reduced fatty acid biosynthesis leading to lower lipid accumulation may address insulin resistance [41]. Amino acids play a direct role in the creation of new glucose through the process of gluconeogenesis [42]. Decreased expression of amino acid biosynthesis enzymes may contribute to improved glycemic control by reducing gluconeogenesis. In addition to the variability in degree of polymerization (DP) observed for isomalto-oligosaccharides (IMOs) derived from the *Bacillus subtilis* strain, it is important to consider the influence of chain length and branching on fermentation characteristics within the intestines. For instance, shorter-chain carbohydrates like inulin-type fructans (ITF) with DP < 10 are known to undergo rapid fermentation in the distal small intestine or proximal colon, whereas longer-chain carbohydrates like inulin may be fermented more slowly, highlighting the complexity of carbohydrate metabolism in the gut. More research is needed to compare fermentable carbohydrates and choose the best prebiotics for gut and metabolic health.

## Conclusions

In summary, to simulate Type 2 diabetes in rats, a high-fat diet along with small doses of streptozotocin was administered in this study. These rats showed similar changes to diabetic patients, including high glucose levels and weight loss. Treatment of the rats with inulin and IMOs resulted in lowered triglyceride levels, potentially through reduced cholesterol absorption and modulation of gene expression. The treatments also improved glucose tolerance, which is beneficial for managing high blood sugar. Additionally, these treatments appeared to reduce inflammation, evidenced by decreased levels of white blood cell and CRP. While further research is warranted, these findings suggest that inulin and IMOs may be beneficial for diabetes and hypercholesterolemia. Changes observed in antioxidant enzymes and metabolic pathways further support this notion. In conclusion, inulin and IMOs show promise for the management of diabetes and associated complications.

### Supplementary Information

Supplementary Material 1: **Figure 1.** a Pictorial representation of the animal experimental design. Following acclimatization, 84 male rats were randomly allocated into two groups of the normal diet group (n = 12), which continued on a normal diet throughout the study, and the high-fat diet group (n = 72), which received a high-fat diet for 14 days. Subsequently, the high-fat diet group was further subdivided into two subgroups of a high-fat diet group (n = 12) and a diabetes group (n = 60). The 60 diabetic rats were induced with 2 doses of STZ. The diabetic rats were then divided into five groups: the diabetic control group (DM; n = 12), the diabetic group supplemented with 1% inulin (DM+1% inulin; n = 12), the diabetic group supplemented with 3% inulin (DM+3% inulin; n = 12), the diabetic group supplemented with 1% IMOs (DM+1% IMOs; n = 12) and the diabetic group supplemented with 3% IMOs (DM+3% IMOs; n = 12).

## Data Availability

Data availability is ensured when corresponding authors are queried.

## Abbreviations

AST: Aspartate transaminase
AUC: Area under the curve
CRP: C-reactive protein
ELISA: Enzyme-linked immunosorbent assay
GO: Gene ontology
HDL: High density lipoprotein
LDL: Low density lipoprotein
SCFA: Short-chain fatty acids
VLDL: Very low-density lipoproteins
