# Supplementary figures and images for: Assessing the therapeutic potential of long-chain isomaltooligosaccharides in diabetic and hyperlipidemic rats
Source: Diabetol Metab Syndr. 2024 Jul 16;16:165. doi: 10.1186/s13098-024-01374-0 (PMC11253390; doi:10.1186/s13098-024-01374-0)

## Slide 1
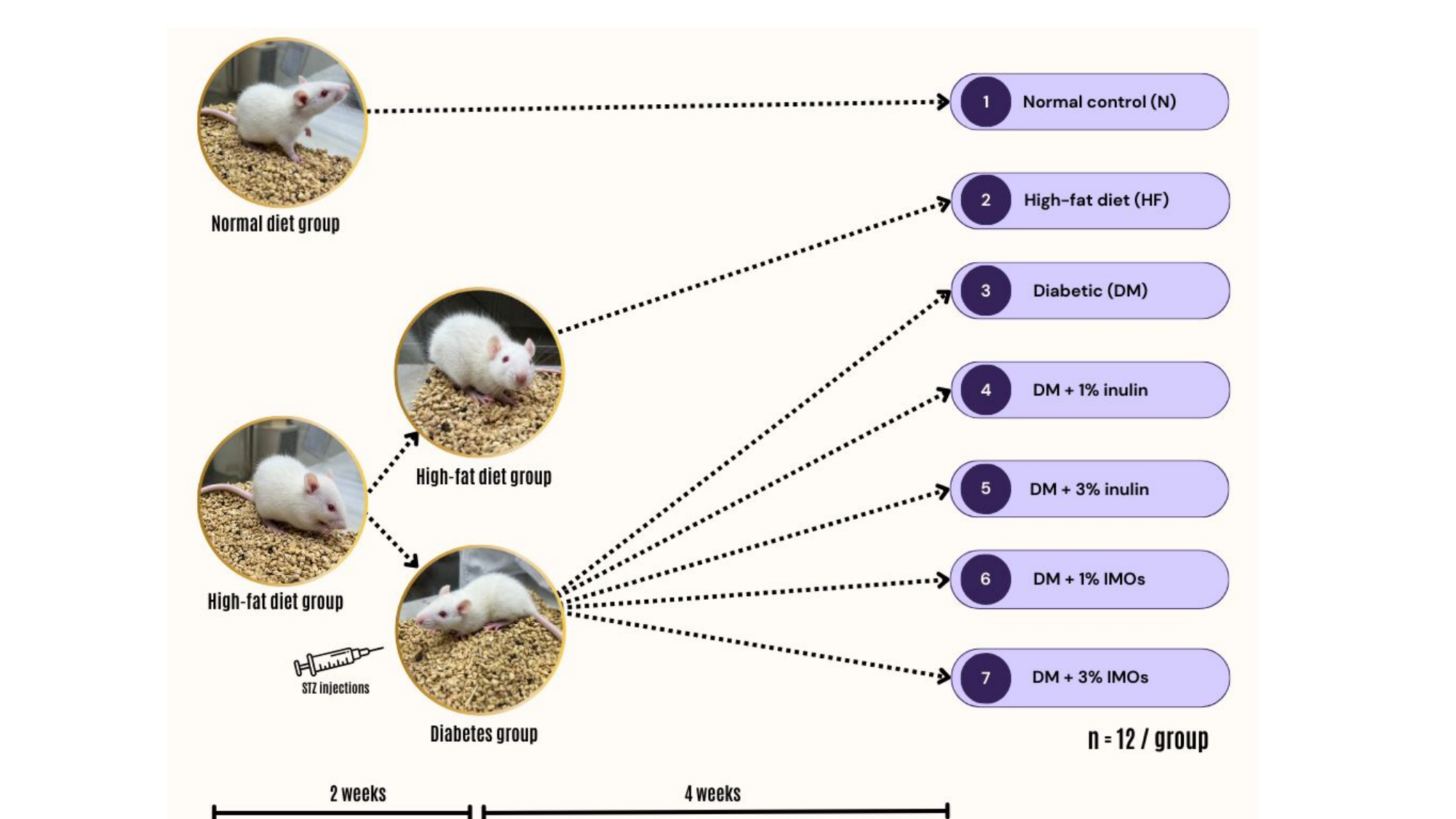

#

Supplement: Supplementary file 1 — Supplementary Material 1: Figure 1. a Pictorial representation of the animal experimental design. Following acclimatization, 84 male rats were randomly allocated into two groups of the normal diet group (n = 12), which continued on a normal diet throughout the study, and the high-fat diet group (n = 72), which received a high-fat diet for 14 days. Subsequently, the high-fat diet group was further subdivided into two subgroups of a high-fat diet group (n = 12) and a diabetes group (n = 60). The 60 diabetic rats were induced with 2 doses of STZ. The diabetic rats were then divided into five groups: the diabetic control group (DM; n = 12), the diabetic group supplemented with 1% inulin (DM+1% inulin; n = 12), the diabetic group supplemented with 3% inulin (DM+3% inulin; n = 12), the diabetic group supplemented with 1% IMOs (DM+1% IMOs; n = 12) and the diabetic group supplemented with 3% IMOs (DM+3% IMOs; n = 12). [file 13098_2024_1374_MOESM1_ESM.pptx]
